# Supplementary material for: Analysis of COVID-19 prevention and treatment in Taiwan
Source: Biomedicine (Taipei). 2021 Mar 1;11(1):1–18. doi: 10.37796/2211-8039.1185 (PMC8823471; doi:10.37796/2211-8039.1185)
Supplement: Supplementary file 1 [file bmed-11-01-001-s001.pdf]

REVIEW ARTICLE

# Analysis of COVID-19 prevention and treatment in Taiwan

Yu-Jen Chiu [a,b,1], Jo-Hua Chiang [c,1], Chih-Wei Fu [d], Mann-Jen Hour [e], Hai-Anh Ha [e,f], Sheng-Chu Kuo [e], Jen-Jyh Lin [g], Ching-Chang Cheng [h], Shih-Chang Tsai [i], Yu-Shiang Lo [j], Yu-Ning Juan [j], Yih-Dih Cheng [e], Jai-Sing Yang [j,**], Fuu-Jen Tsai [k,l,*]

[a] Division of Plastic and Reconstructive Surgery, Department of Surgery, Taipei Veteran General Hospital, Taipei 11217, Taiwan
[b] Department of Surgery, School of Medicine, National Yang Ming University, Taipei 11217, Taiwan
[c] Department of Nursing, Chung-Jen Junior College of Nursing, Health Sciences and Management, Chiayi County 62241, Taiwan
[d] Biomedical Technology and Device Research Laboratories, Industrial Technology Research Institute, Hsinchu 310401, Taiwan
[e] School of Pharmacy, China Medical University, Taichung 40402, Taiwan
[f] Faculty of Pharmacy, Duy Tan University, Da Nang 550000, Viet Nam
[g] Division of Cardiology, Department of Medicine, China Medical University Hospital, Taichung 404, Taiwan
[h] Laboratory Animal Service Center, China Medical University, Taichung 40402, Taiwan
[i] Department of Biological Science and Technology, China Medical University, Taichung 40447, Taiwan
[j] Department of Medical Research, China Medical University Hospital, China Medical University, Taichung 40447, Taiwan
[k] School of Chinese Medicine, College of Chinese Medicine, China Medical University, Taichung 40402, Taiwan
[l] China Medical University Children's Hospital, China Medical University, Taichung 40402, Taiwan

### Abstract

**Coronavirus disease 2019 (COVID-19) has been spreading worldwide with a mind-boggling speed. According to a statement from World Health Organization (WHO), COVID-19 has infected more than six billions people and caused more than one and half million passing in the world. Based on previous experience with SARS, the Taiwanese government had decided to block viral transmission during its early stages. This review sums up the clinical characteristics, Severe Acute Respiratory Syndrome coronavirus 2 (SARS-CoV-2) viral infection process, diagnostic methods, preventive strategy, and the executive proportions of COVID-19, as well as the name-based mask distribution system (NBMDS) in Taiwan. We also give a review of the conceivable sub-atomic pharmacologic systems against SARS-CoV-2 specialists and the blend of remdesivir (GS-5734), chloroquine (CQ), and hydroxychloroquine (HCQ). Lastly, we summarized the therapeutic agents against COVID-19 as mentioned by COVID-19 treatment guidelines. In this review, development of novel anti-SARS-CoV-2 viral agents, vaccines for COVID-19 therapy or an effective combination therapy can be expected based on all the information accumulated. Last but not least, we might want to stretch out our best respects to all medical providers in their worldwide battle against COVID-19.**

*Keywords:* Coronavirus disease 2019 (COVID-19), Respiratory Syndrome coronavirus 2 (SARS-CoV-2), Name-based mask distribution system (NBMDS), Therapeutic agents, Preventive strategy

## 1. Introduction

Coronavirus disease 2019 (COVID-19), brought about by severe acute respiratory syndrome coronavirus 2 (SARS-CoV-2) infection, has spread worldwide with a mind-boggling speed. According to a statement from World Health Organization (WHO), COVID-19 has

Received 14 December 2020; revised 15 December 2020; accepted 16 December 2020.
Available online 01 March 2021

* Corresponding author at: School of Chinese Medicine, College of Chinese Medicine, China Medical University, 91 Hsueh-Shih Road, Taichung 40402, Taiwan.

** Corresponding author at: Department of Medical Research, China Medical University Hospital, China Medical University, 2 Yude Road, Taichung 40447, Taiwan.
E-mail addresses: jaisingyang@gmail.com (J.-S. Yang), d0704@mail.cmuh.org.tw (F.-J. Tsai).
[1] Both authors contributed equally.

https://doi.org/10.37796/2211-8039.1185
2211-8039/Published by China Medical University 2020. © the Author(s). This is an open access article under the CC BY license (http://creativecommons.org/licenses/by/4.0/).

BioMedicine
2020;11(1):1–18

REVIEW ARTICLE

infected more than six billions people and caused more than one and half million passing in the world [1–6]. Based on previous experience with severe acute respiratory syndrome (SARS), the Taiwanese government had decided to block viral transmission during its early stages. Presently, early blockage of SARS-CoV-2 transmission has been the key point in protecting against COVID-19 [7,8]. Confirmed cases in Taiwan have been lower than those in other countries. Accordingly, the Taiwan Center for Disease Control (TCDC) had revealed 675 affirmed cases and seven passing till the end of November 2020 (Fig. 1A). The majority of the confirmed cases were indigenous and imported, with a peak age of 20-29 years (Fig. 1B).

Taiwan is only 81 miles away from the coast of China, has been constantly alert and ready to act on potential epidemics arising from China considering the insufferable experiences gained from the severe acute respiratory syndrome (SARS) epidemic of 2003 [8]. Given that most patients shared nonspecific clinical and laboratory findings, comprehensive surveillance of detailed exposure history for suspected patients, and application of rapid detection tools are required. Through the combination of border control, rapid testing and quarantine of individuals with contact history, isolation, real-time linking of informative records with the healthcare system, and protection of health care worker safety through Traffic Control Bundling, Taiwan had been able to effectively control the COVID-19 epidemic [9,10].

We sums up the clinical characteristics, SARS-CoV-2 viral life cycle and infection process, diagnostic methods, preventive strategy, and the executive proportions of COVID-19, as well as the name-based mask distribution system (NBMDS) in Taiwan. We also give a review of the conceivable sub-atomic pharmacologic systems against SARS-CoV-2 specialists. Lastly, we summarized the therapeutic agents against COVID-19 as mentioned by COVID-19 treatment guidelines.

## 2. Clinical manifestations of COVID-19

As indicated by literature reviews, fever, weariness, headache, dry cough, sputum production, haemoptysis, sore throat, and diarrhoea have been the most usual manifestations at the beginning of COVID-19 infection, with other symptoms including muscle ache, dyspnoea, productive cough, haemoptysis, and stroke [11]. The time period of

A.

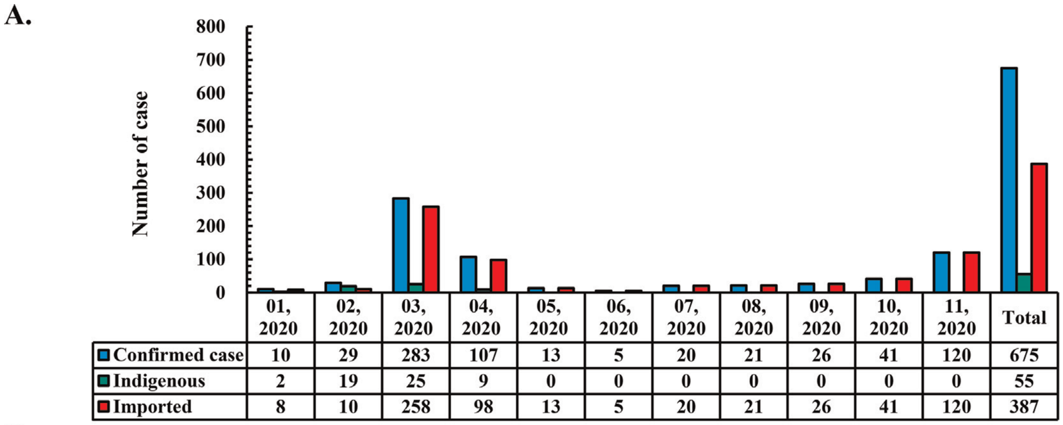


| | 01, 2020 | 02, 2020 | 03, 2020 | 04, 2020 | 05, 2020 | 06, 2020 | 07, 2020 | 08, 2020 | 09, 2020 | 10, 2020 | 11, 2020 | Total |
|---|---|---|---|---|---|---|---|---|---|---|---|---|
| Confirmed case | 10 | 29 | 283 | 107 | 13 | 5 | 20 | 21 | 26 | 41 | 120 | 675 |
| Indigenous | 2 | 19 | 25 | 9 | 0 | 0 | 0 | 0 | 0 | 0 | 0 | 55 |
| Imported | 8 | 10 | 258 | 98 | 13 | 5 | 20 | 21 | 26 | 41 | 120 | 387 |

B.

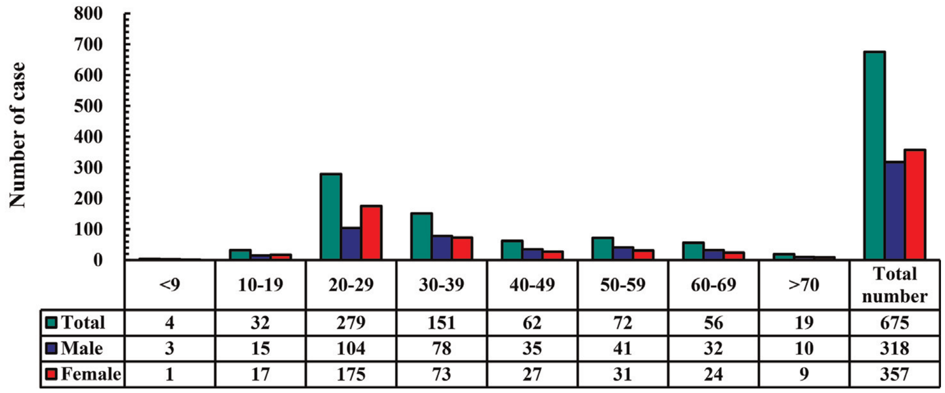


| | <9 | 10-19 | 20-29 | 30-39 | 40-49 | 50-59 | 60-69 | >70 | Total number |
|---|---|---|---|---|---|---|---|---|---|
| Total | 4 | 32 | 279 | 151 | 62 | 72 | 56 | 19 | 675 |
| Male | 3 | 15 | 104 | 78 | 35 | 41 | 32 | 10 | 318 |
| Female | 1 | 17 | 175 | 73 | 27 | 31 | 24 | 9 | 357 |

*Fig. 1. Confirmed cases in Taiwan. (A) Number of confirmed cases of coronavirus (COVID-19) in Taiwan till the end of November 2020. (B) The numbers are divided into several 10-year age groups.*

symptoms onset is around 5.2 days after infection [11]. It was reported that up to 75% of patients with COVID-19 infection stay asymptomatic [11,12]. Around 14% of patients develop severe symptoms and signs, including pneumonia and adult respiratory distress syndrome (ARDS), requiring hospitalization and oxygen treatment, and about 5% of patients need intensive care and support. From admission to intensive care unit, the time period of length was around 10 days, while the span between manifestation beginning and passing went from roughly 2 weeks to 2 months [11,13,14]. Fig. 2 revealed the symptoms of signs as COVID- 19 infection progressing.

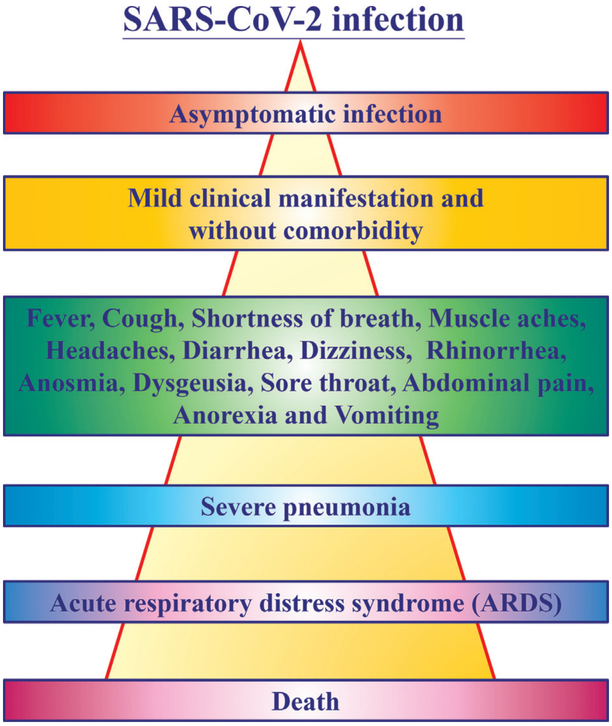


*Fig. 2. Symptoms of the COVID-19.*

Finding of laboratory tests incorporate raised lactate dehydrogenase and ferritin levels. Leucopoenia and lymphopenia have been most ordinarily noticed discoveries, while white blood cell (WBC) counts can vary [15–17]. Findings of chest radiography and computed tomography (CT) are assorted and vague, regularly introducing as local patchy shadowing, bilateral patchy shadowing or multiple ground-glass opacity lesions. As the infection advances, ground-glass opacity lesions might advance into consolidation or crazy-paving pattern finding (interlobular/intralobular septal thickening) [18–20].

## 3. Diagnostic testing of the COVID-19 in Taiwan

Diagnosis tests of COVID-19 in Taiwan were summarized in Table 1. There are three methods which have been popularly applied for the diagnosis of SARS-CoV-2, including:

(1) A nucleic acid amplification test (NAAT) by real-time reverse transcription polymerase chain reaction (rRT-PCR).
(2) Antigen test screening.
(3) Serologic and/or antibody testing.

A nucleic acid amplification test (NAAT) by real-time reverse transcriptase polymerase chain reaction (rRT-PCR) is the gold standard for detecting SARS-CoV-2 infection. The specificity and sensitivity are very high. However, this method has a window period of up to 5 days after virus exposure.

*Table 1. Diagnosis of COVID-19 in Taiwan [21–24].*

| Types | Methods | Detection |
|---|---|---|
| SARS-CoV-2 commercial test system (Roche, cobas® SARS-CoV-2). | Real-time reverse transcriptase PCR (rRT-PCR) test intended for the qualitative detection of nucleic acids from SARS-CoV-2 in nasopharyngeal and oropharyngeal swab samples | Viral nucleic acids<br>FDA approved COVID-19 test Kit |
| Rapid screening and serologic diagnosis | Lateral flow immunoassays<br>Time-resolved fluorescence immunoassay | Detect the presence of IgG and IgM from whole blood, serum or plasma |
| Hematology and biochemistry examination | Cell counter and biochemistry reaction Kit | 1. White blood cell count <4 × $10^9$/L Lymphocyte count <1 × $10^9$/L<br>2. C-reactive protein (CRP) level<br>3. Lactate dehydrogenase (LDH)<br>4. Aspartate aminotransferase (AST)<br>5. Alanine aminotransferase (ALT),<br>6. Creatine kinase (CK)<br>7. D-dimer (a fibrin degradation product present in the blood) |
| Nucleic Acid | 1. Next generation sequencing platforms (NGS) | Viral sequence |
| Imaging technology | 1. Chest radiograph<br>2. CT images | 1. Bilateral distribution of patchy shadows<br>2. Ground glass opacity |

FDA: U.S. Food and Drug Administration; IgG: Immunoglobulin G; IgM: Immunoglobulin M; CT: computed tomography.

Antigen tests can rapidly identify people with SARS-CoV-2 infection with low cost, which is critical for preventing transmission. However, low accuracy is the major problem of antigen test. A nucleic acid amplification test (NAAT) should be considered, if a person who is highly suspected of SARS-CoV-2 infection with negative results of initial antigen tests. Serologic and/or antibody testing is not suit for diagnosing initial SARS-CoV-2 infection, because these antibodies to SARS-CoV-2 were merged after 21 days or more after infection. But, the test can investigate people with prior or recent SARS-CoV-2 infection (Fig. 3) [21—24].

## 4. Structure and life cycle of SARS-CoV-2

Both SARS-CoV-2 and Middle East Respiratory Syndrome Coronavirus (MERS-CoV) have been considered profoundly pathogenic. As shown in Fig. 4A, the schematic structure of SARS-CoV-2 included spike proteins (S), lipid membrane, membrane proteins (M), envelope proteins (E), and nucleocapsid protein enclosing ssRNA (N). Single stranded RNA genome of SARS-CoV-2 was shown in Fig. 4B [25]. The SARS-CoV-2 use their spike proteins to bind host cell membrane receptors, angiotensin-converting enzyme 2 (ACE2) and transmembrane serine protease 2 (TMPRSS2), for passage. Fig. 5 shows that there are eight significant stages of the life cycle of SARS-CoV-2. Stage 1 is that SARS-CoV-2 enters the target cell through endocytosis or fusion after authoritative to ACE2 receptor and TMPRSS2. The SARS-CoV-2 envelope advances viral RNA genome discharge into the host cell cytoplasm through fusing with the endosome layer in the lysosomal corrosive condition. Subsequently, stage 2 is genome RNA release. The coronavirus main proteinase (3CLpro) is deciphered for the replication of genomic RNA. From that point, replicase polypeptide is proteolysis, delivering helicase and RNA-dependent RNA polymerase (RdRp) (stage 3 and 4). In stage 5, SARS-CoV-2 at that point goes through viral RNA replication in the host cells. In stage 6, the viral sub-genome is deciphered. Spike proteins (S), lipid membrane, membrane proteins (M), envelope proteins (E), and nucleocapsid protein enclosing ssRNA (N) are packaging through Golgi apparatus and the endoplasmic reticulum (stage 7). In stage 8, a novel virion is packed and shaped by N protein and other auxiliary proteins communicate with viral genomic RNA. Finally, the amassed virion is then delivered by means of exocytosis into the extracellular compartment [11,26,27].

## 5. Preventive strategy of COVID-19 in Taiwan

SARS-CoV-2 infection possesses nonspecific clinical manifestation, so that it can be easily transmitted from mild symptomatic or asymptomatic individuals to others. As of now, avoidance of viral passage into the human body seems to be the most ideal choice for controlling viral spread. The Taiwan Center for Disease Control (TCDC) has set up specialized rules for COVID-19. Coming up next are vital points for forestalling viral spread:

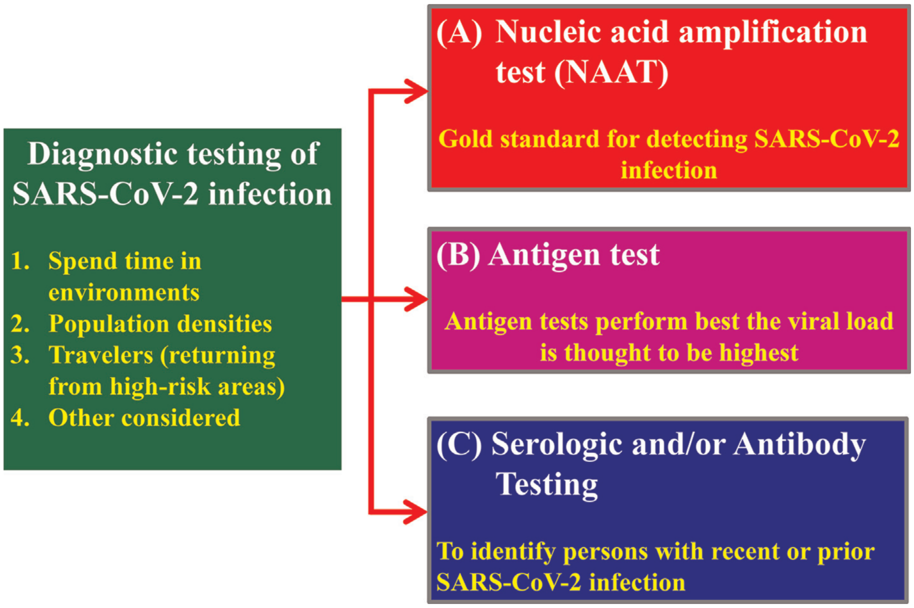


*Fig. 3. Diagnostic testing of the COVID-19.*

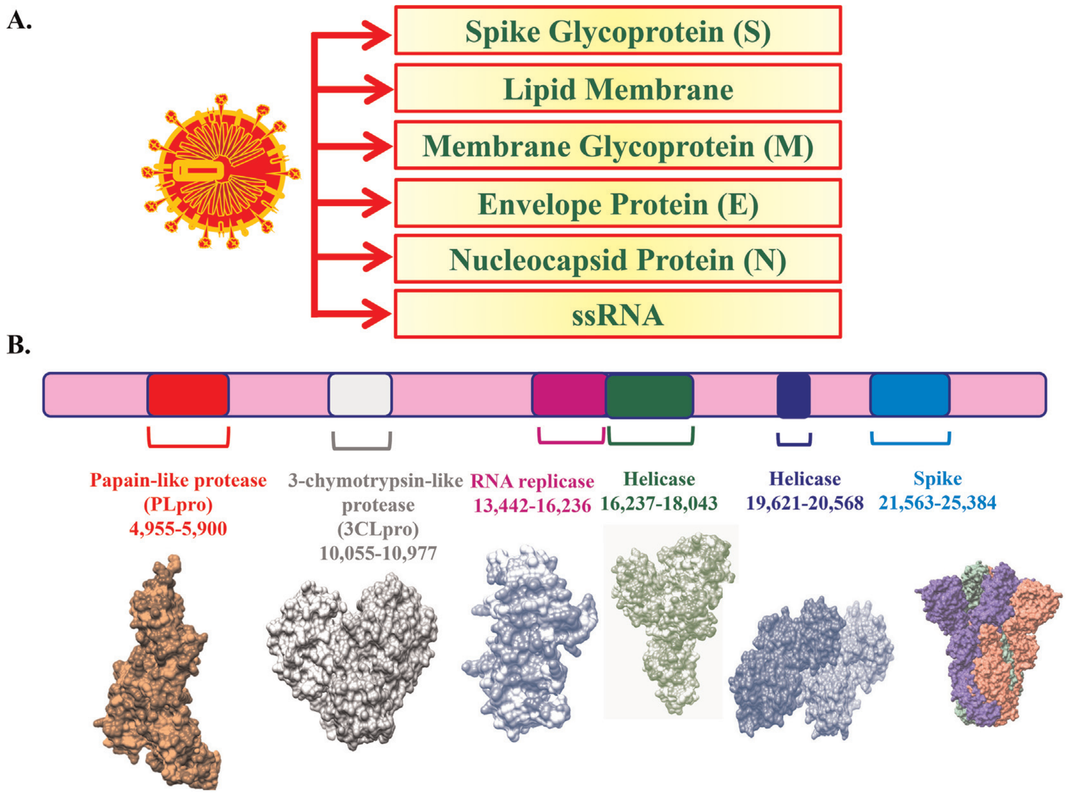


*Fig. 4. The protein structure and RNA genome of SARS-CoV-2. (A) The major structural proteins including the spike glycoprotein (S), membrane glycoprotein (M), envelope protein (E) and Nucleocapsid Protein (N) on SARS-CoV-2. (B) Single stranded RNA genome of SARS-CoV-2.*

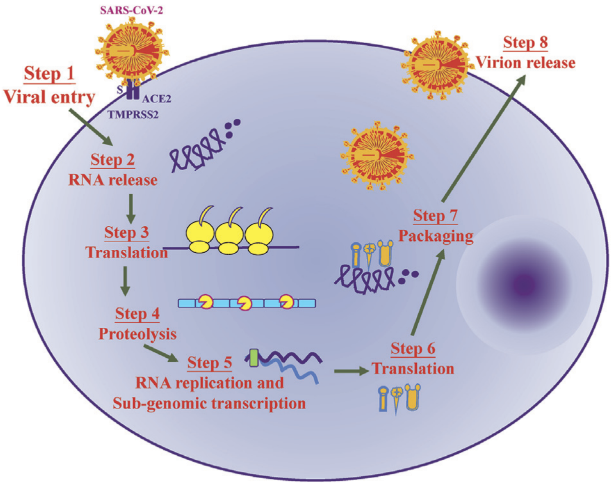


*Fig. 5. SARS-CoV-2 life cycle. Stages of the SARS-CoV-2 life cycle include virus entry, RNA release, translation, proteolysis, RNA replication and sub-genomic transcription, translation, packaging and virion release.*

BioMedicine
2020;11(1):1–18



(1) Avoiding travelling to affected countries.
(2) Regular decontamination with 5% to 10% sodium hypochlorite.
(3) Social distance, including at least 1.5 m indoors and at least 1 m outdoors.
(4) Keeping proper hygiene of individuals, such as frequent hand washing with soap or alcohol-based hand sanitisers.
(5) Personal protective equipment, such as gloves, eye protection, gowns and medical masks [28].

Because of the supply shortages of face masks, The Taiwanese government has developed guidelines to protect the health and safety of the public from the global novel coronavirus outbreak. Fig. 6 details the processing of the name-based mask distribution system (NBMDS) in Taiwan [29]. While medical and surgical masks should be prioritised for health care workers, the general public can wear cloth face masks made from household items, such as two layers of cotton fabric, T-shirts or bedsheets. Medical masks can filter 5-μm particles from the air reaching the mouth/nose, reducing the transmission of respiratory droplets to others and prevent blood or other potentially infectious materials from reaching the wearer's skin, mouth or mucous membranes. Fig. 7 summarise the medical mask materials and associated principles [29–34]. In addition, the structure and composition of the different virus families occur affect their reaction to disinfectants. Components, such as 75% ethanol [35–38], sodium hypochlorite (1000 ppm (0.1%)-10,000 ppm (1%)) [33,38,39], hypochlorous acid (10 ppm-30 ppm) [33,38–40], chlorine dioxide [40], soap [41] and hydrogen peroxide (0.5%) [42–44] and others [44–47] have been used to kill bacteria and viruses. Table 2 lists the chemical formula and preparation concentration of the disinfectants, as well as associated principles.

## 6. Current therapeutic modalities for COVID-19 in Taiwan, and summary of therapeutic agents against COVID-19 as mentioned by COVID-19 treatment guidelines

Currently, there is still lacking strong clinical evidence of existing anti-viral agents and vaccine against SARS-CoV-2 infection. Supportive treatment is crucial, including oxygen therapy for hypoxemia and respiratory distress, intravenous fluids

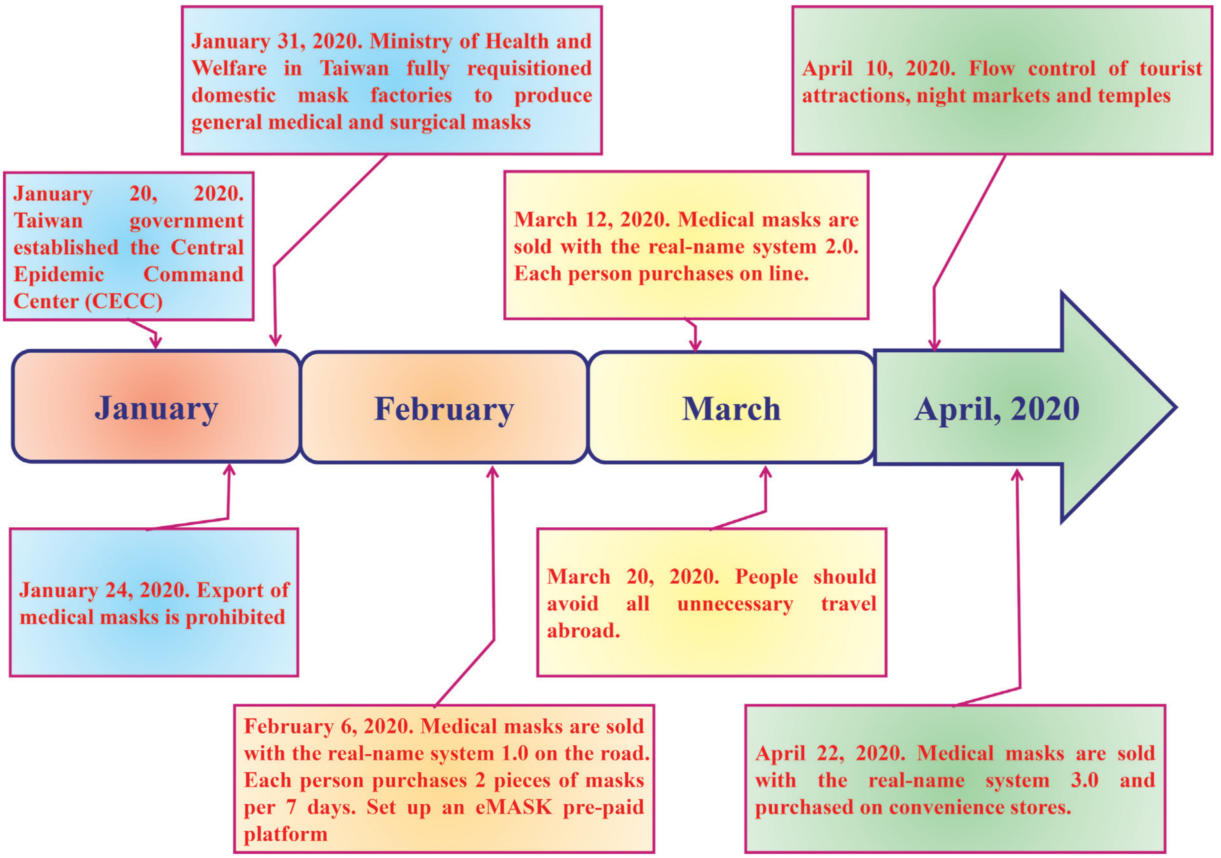


*Fig. 6. Policies for controlling mask distribution and mask wearing to prevent viral transmission in Taiwan.*

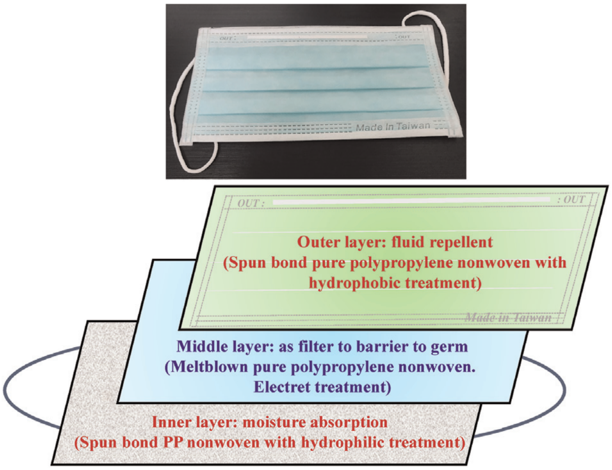


*Fig. 7. Design of the three-layer non-medical face masks for the protection of the general public against viral infection. The three-layer material is made from pure polypropylene melt-blown polymer (middle layer), placed between two non-woven fabric layers. The outer layer is fluid repellent, while and inner layer absorbs moisture.*

*Table 2. Possible preventive methods of SARS-CoV-2 infection [35–38,40,44,47].*

| Methods | Principles | Chemical formula | Concentration |
|---|---|---|---|
| Medical mask | A medical/surgical mask help people to protect users from large respiratory droplets of patients. Three ways of removing particles from the airstream, such as (i) inertial impaction, (ii) diffusion, and (iii) electrostatic attraction. This mask has three-layer materials made up of a melt-blown polymer (most commonly polypropylene) that prevents microbes from entering the mask A minimum of bacterial filtration efficiency (BFE) of the medical mask is over 95% filtration rate. | Non | Non |
| 75% ethanol | Ethanol is a volatile, flammable and naturally produced through petrochemical processes. Its effects on lipid in bacterial cell wall and cell membrane can lead to protein denaturation. | $CH_3CH_2OH$ | 70%~75% |
| Sodium hypochlorite | Hypochlorous acid produced by the reaction of sodium hypochlorite (NaClO) with carbon dioxide is a component of bleach. It is a strong oxidant, in the form of gas or in combination with other chemicals. | NaClO | 1000 ppm (0.1%) ~ 10,000 ppm (1%) |
| Hypochlorous acid | Hypochlorous acid (HClO) is a weak acid from that chlorine dissolves in water. It is the simplest oxo-acid of chlorine which is involved in fast equilibria with oxidizers (hypochlorite, HClO and $ClO^-$) under acidic conditions. Hypochlorous acid is effective at killing viruses. | HClO | 10 ppm ~30 ppm |
| Chlorine dioxide | Chlorine dioxide ($ClO_2$) is an inorganic compound from oxygen and chloride of two electronegative elements. This compound property makes possible to exhibit the action as an anti-microbial agent. | $ClO_2$ | 0.03 ppm ~0.10 ppm |
| Soap | They are amphiphilic: partly hydrophilic (polar) and partly hydrophobic (non-polar). Their dual nature facilitates the mixture of hydrophobic compounds (like oil and grease) with water. Break the oil structure can form small fragments (emulsification). | Non | Non |
| Hydrogen peroxide | An oxidizing agent has the oxidizing ability. Common oxidizing agents is hydrogen peroxide (0.5%). Oxidize cell contents to locally inactivate. | $H_2O_2$ | 0.5% |
| Other | Quaternary ammonium compounds. | Non | Non |

support, and ventilator and extracorporeal membrane oxygenation therapy for patients with adult respiratory distress syndrome (ARDS). Patients with severe ARDS may benefit from systemic corticosteroids. Supportive treatments are summarized in Table 3 [48,49]. Currently, three clinical trials on COVID-19 are ongoing in Taiwan (NCT 04292899, NCT 04292730 and NCT 03808922), with remdesivir (Veklury®) as the primary anti-SARS-CoV-2 agent.

In the one-year period since the epidemic arose, scientists around the world made many efforts to find ways to prevent and treat the disease [11]. Based on the accumulating data, guidelines on COVID-19 treatment is gradually being updated and modified by the World Health Organization (WHO), United States National Institutes of Health (NIH) as well as other Health authorities [1—5]. Categorically, agents used for COVID-19 can be roughly divided into 3 main groups (Fig. 8):

(A) Anti-viral therapy agents.
(B) Immune-based therapy agents.
(C) Adjunctive therapy agents.

*Table 3. Supportive therapy for clinical conditions in COVID-19 [48,49].*

| Symptoms | Treatments | Targets and notes |
|---|---|---|
| Patients with respiratory distress, hypoxemia or shock | 1. Give supplemental oxygen therapy<br>2. Initiate oxygen therapy at 5 L/min and titrate flow rate accordingly<br>3. Intravenous fluids support | 1. Non-pregnant patients: $SpO_2 \geq 90\%$<br>2. Pregnant patients: $SpO_2 \geq$ 92-95%<br>3. Children without emergency signs: $SpO_2 \geq 90\%$<br>4. Children with red flag signs: $SpO_2 \geq 94\%$,<br>5. Patients treated with intravenous fluids cautiously, and since aggressive fluid resuscitation may worsen oxygenation |
| Patients with severe ARDS | 1.Ventilator support combined with/without extracorporeal membrane oxygenation therapy.<br>2. systemic corticosteroids | 1. Chest images presented as pneumonia. Oxygenation impairment: with the minimum level of PEEP 5cmH2O, PaO2/FiO2 ratio ≤300 and > 200 is mild ARDS; PaO2/FiO2 ratio 100- 200 is moderate ARDS; PaO2/FiO2 ratio <100 is severe ARDS.<br>2. Patients with severe ARDS may benefit from systemic corticosteroids. |

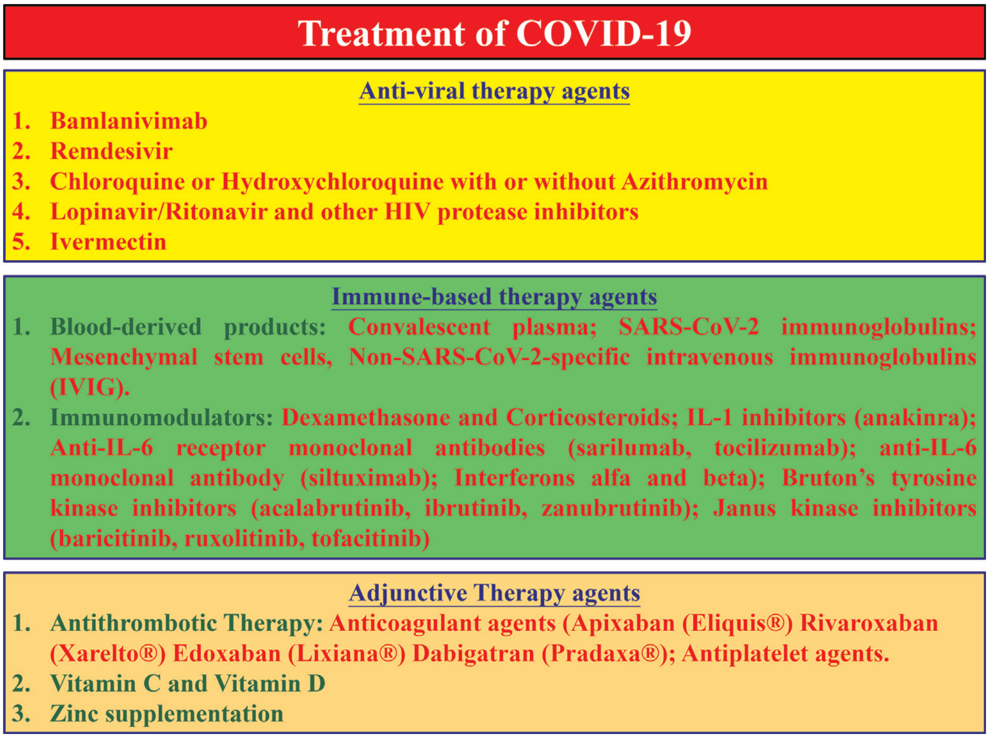


*Fig. 8. Overview of therapy agents on COVID-19.*

In the latest update of NIH's guideline (updated on Nov 18th, 2020), bamlanivimab (LY-CoV555) is the new therapy to fight against COVID-19. This synthetic antibody-based treatment is received Emergency Use Authorization, but NIH recommended that is not a standard care for COVID-19 [2]. It can be used for non-hospitalized patients with mild to moderate COVID-19 and having high risk of progressing to severe condition. Earlier, remdesivir received approval from Europe Commission and US-FDA, become the first official drug to treat COVID-19 [1,2]. Lopinavir/ritonavir (LPV/r; Kaletra®) and ivermectin (Stromectol®) were proposed as potential therapeutic for COVID-19 in early 2020 [50]. However, the limited clinical improvement, no significant in lowering morality rate, and made these therapeutics got recommendation against by the guidelines, except for clinical trials. Other treatments of anti-viral therapies can be found at China's guideline (the latest updated on Aug 18th, 2020) with the recommendation of using chloroquine sulfate (CQ) and ribavirin (Robatrol®) combined with interferon α or lopinavir/ritonavir (LPV/r; Kaletra®) [4,51]. Also, in this guideline, the uses of traditional medicine were recommended for treatment of COVID-19. The distinctive, different opinions of China's guideline compared to others, and the accomplishments in controlling disease of China are worth to be analyzed (Table 4). However, comprehensive clinical data on that the achievements were not published so far.

Immune-based therapies for COVID-19 are considered as systemic treatments, using human blood-derived products and/or immune-modulatory therapies. As of blood-derived products, those who have recovered from SARS-CoV-2 infection can donate their convalescent plasma or immunoglobulin to make products for treatment in other patients [52,53]. Experimental data suggested that, these products induce direct inhibitory effect against SARS-CoV-2 [53]. There is not enough evidence in clinical data for blood-derived products to be recommended by the guidelines as a standard care treatment for COVID-19 [2,3,5]. Other therapies, such as mesenchymal stem cells, non-SARS-CoV-2-specific intravenous immunoglobulins (IVIG) are recommended against by experts to use for COVID-19, except in clinical trial [2]. As of the corticosteroids, the most recommended agent in guidelines is dexamethasone, based on the accumulated data that showed the improvement in clinical treatment for COVID-19 patients. However, the guidelines recommended against the long-term use of dexamethasone and other corticosteroids, due to the side effects [1–5]. In addition, the combination of dexamethasone and remdesivir is suggested by the NIH's guideline [2]. Other immunmodulators (IL-1 inhibitors; anti-IL-6 receptor monoclonal antibody; Interferons α/β; Bruton's tyrosine kinase inhibitors and Janus kinase inhibitors) are mentioned cautiously by the guidelines. Due to the lack of supportive evidence, these products are not recommended for use in patients with COVID-19, except in well-designed trials [2,3,5].

Adjunctive therapy agents for use in COVID-19 course are recommended by the guidelines, including:

(1) Anti-thrombotic therapies.
(2) Vitamin C and vitamin D.
(3) Zinc supplementation.

Because the symptoms of COVID-19 are associated with inflammation and a pro-thrombotic state, anti-thrombotic agents and anti-platelet agents were suggested as useful adjunct to COVID-19 treatment [2,5]. Recommendations for anti-thrombotic agents were given by the guidelines, such as apixaban (Eliquis®), rivaroxaban (Xarelto®), edoxaban (Lixiana®) and dabigatran (Pradaxa®) (by the NIH - USA) and acetylsalicylic acid (Aspirin®) (by NCCET-Australia and NHC-China). However, additional measures should be taken to prevent the possible unwanted effect for certain patient groups and risk/benefit should be considered [5]. Additionally, the uses vitamin and mineral supplements were suggested as a preventive measure for COVID-19, as well as for attenuating the complications during course of the infection. The products of vitamin C, vitamin D and zinc supplementations were suggested by several studies. However, the rationale use, benefits and harms in particular group of patients were need further study to be clarified [1,2,5].

Please refer to the previously published article "Approaches towards fighting the COVID-19 pandemic (Review)" for molecular pharmacological mechanisms in details [11].

## 7. Introduction of synthesis methods on remdesivir (Veklury®), chloroquine (CQ) and hydroxychloroquine (HCQ)

Remdesivir (Compound 12) was synthesised by Siegel et al. as illustrated in Schem 1 (Fig. 9) [54]. The iodo-based compound 1 was reacted with Turbo Grignard reagents via metal–halogen exchange, followed by the addition of ribolactone 2 to afford the glycosylation product 3. Treatment of 3

*Table 4. Summary of anti-viral agents against SARS-CoV-2 of COVID-19 by guidelines.*

| Anti-viral agents for COVID-19 | Pharmacologic mechanisms | Guidelines | | | | |
|---|---|---|---|---|---|---|
| | | WHO (Nov 20th 2020) | NIH (USA) (Nov 18th 2020) | HCSP (France) (Oct 19th 2020) | NHC (China) (Aug 18th 2020) | NCCET (Australia) (Nov 26th 2020) |
| Bamlanivimab (LY-CoV555) | 1. Targets the receptor-binding domain of the spike protein | Not mentioned | Recommended against | Not mentioned | Not mentioned | Not mentioned |
| Chloroquine (CQ) (Aralan®) | 1. Inhibition of viral fusion | Recommended against | Recommended against | Recommended against | Recommended | Recommended against |
| Hydroxychloroquine (HCQ) (Plaquenil®) | | Recommended against | Recommended against | Recommended against | Recommended against | Recommended against |
| Umifenovir (Arbidol®) | 1. Inhibition of viral fusion | Recommended against | No recommendation | Recommended against | Recommended | Recommended against |
| Remdesivir (GS-5734; Veklury®) | 1. Inhibits RNA-dependent RNA polymerase (RpRd) | Recommended against | Conditional recommended with or without corticosteroids | Conditional recommended | No recommendation | Conditional recommended for patients require oxygen but not ventilation |
| Favipiravir (Avigan®) | 1. Inhibits RNA-dependent RNA polymerase (RpRd) | Recommended against | Not mentioned | Recommended against | Not mentioned | Recommended against |
| Lopinavir/ritonavir (Kaletra®) | 1. Inhibits 3C-like protease (3CLpro) | Recommended against | Recommended against | Recommended against | Recommended against monotherapy | Recommended against |
| Ivermectin (Stromectol®) | 1. Inhibits protease | Not mentioned | Recommended against | Not mentioned | Not mentioned | Recommended against |
| Ribavirin (Rebetol®) | 1. Inhibits RNA-dependent RNA polymerase | Not mentioned | Not mentioned | Recommended against | Recommended to use with interferon $\alpha$ or lopinavir/ritonavir | Not mentioned |

Abbreviations: WHO: World Health Organization; NIH: United States National Institutes of Health; HCSP: Haut Conseil de la santé publique; NHC: China's National Health Commission; NCCET: Australian National COVID-19 Clinical Evidence Taskforce.

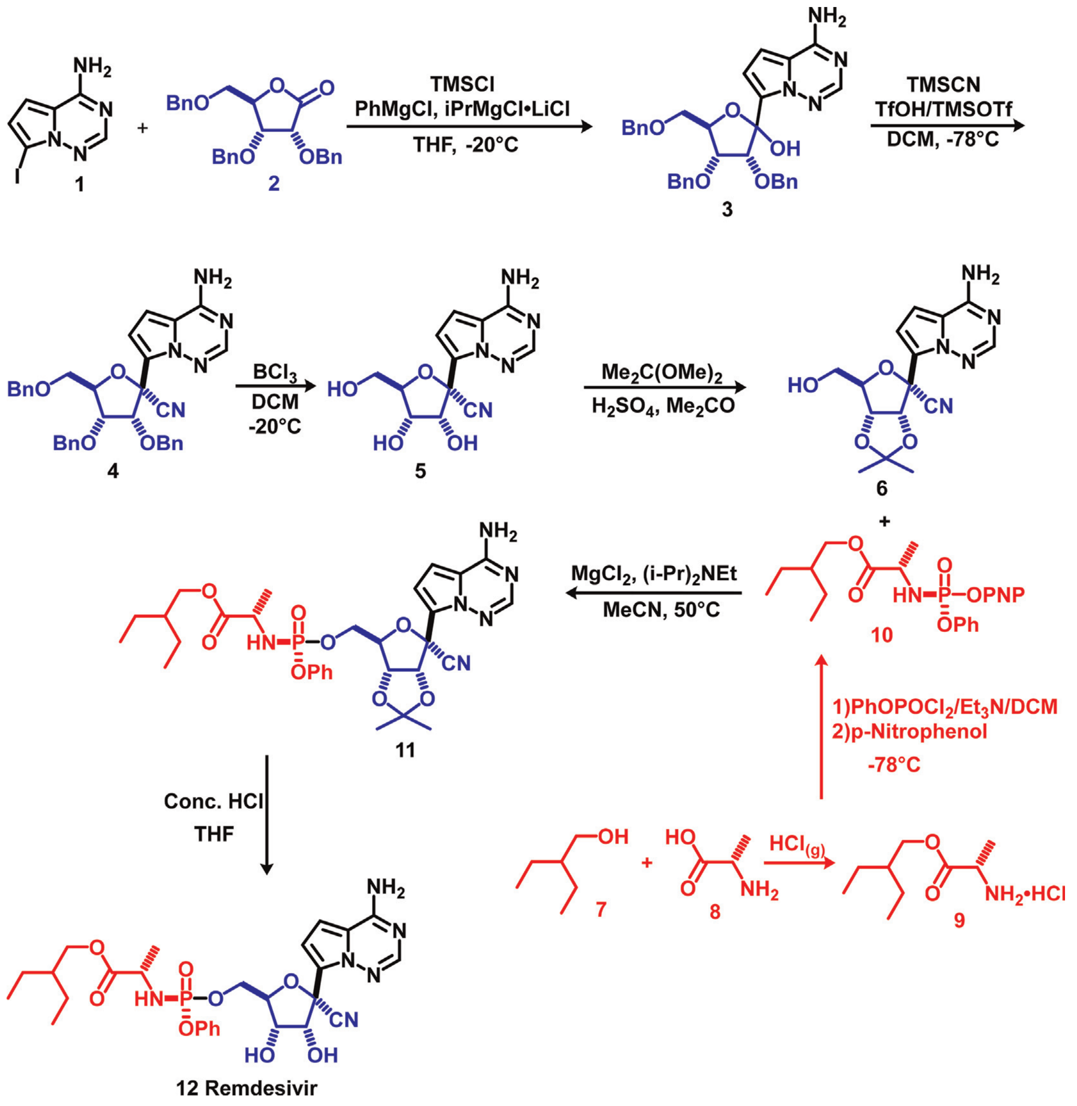


*Fig. 9. Synthesis of remdesivir.*

with TMSCN, TMSOTf and TfOH at −78 °C afforded 4, which yielded benzyl deprotection product 5 after reacting with $BCl_3$. Acetonide protection of the 2′,3′-hydroxyl moieties with 2,2-dimethoxypropane in the presence of $H_2SO_4$ afforded 6. 2-Ethyl-1-butanol 7 and L-alanine 8 were treated with $HCl_{(g)}$ to generate ester product 9, which was reacted with $OP(OPh)Cl_2$ under base conditions, followed by 4-nitrophenol to obtain the *p*-nitrophenolate 2-ethylbutyl-L-alaninate prodrug precursor 10. The coupling reaction between 6 and 10 under $MgCl_2$ generated 11, after which in situ acetonide deprotection was performed through concentrated HCl to afford target molecule remdesivir (Compound 12). Molecular docking of remdesivir binding to the RNA-dependent RNA polymerase (RdRp) was shown on Supplementary video S1 (https://youtu.be/s_SUUMO7URw).

As shown in Fig. 10, chloroquine (Compound 25) was synthesised by Drake N. L. et al. and Price Ch. C. et al. as described in Schem 2 [55,56]. Accordingly, 4,7-Dichloroquinoline 19 was prepared from 3-chloroaniline 13 via 1,4-addition with ethoxymethylenmalonic acid 14, thermal heterocyclisation, hydrolysis, decarboxylation and $POCl_3$ chlorination. Novaldiamine 24 was synthesised following three steps. Acetoacetic ester 20 alkylation with 2-diethylaminoethylchloride 21 generated 2-diethylaminoethylacetoacetic acid ester 22, which yielded 1-diethylamino-4-pentanone 23 upon acidic hydrolysis using hydrochloric acid and simultaneous decarboxylation. Reductive amination of this

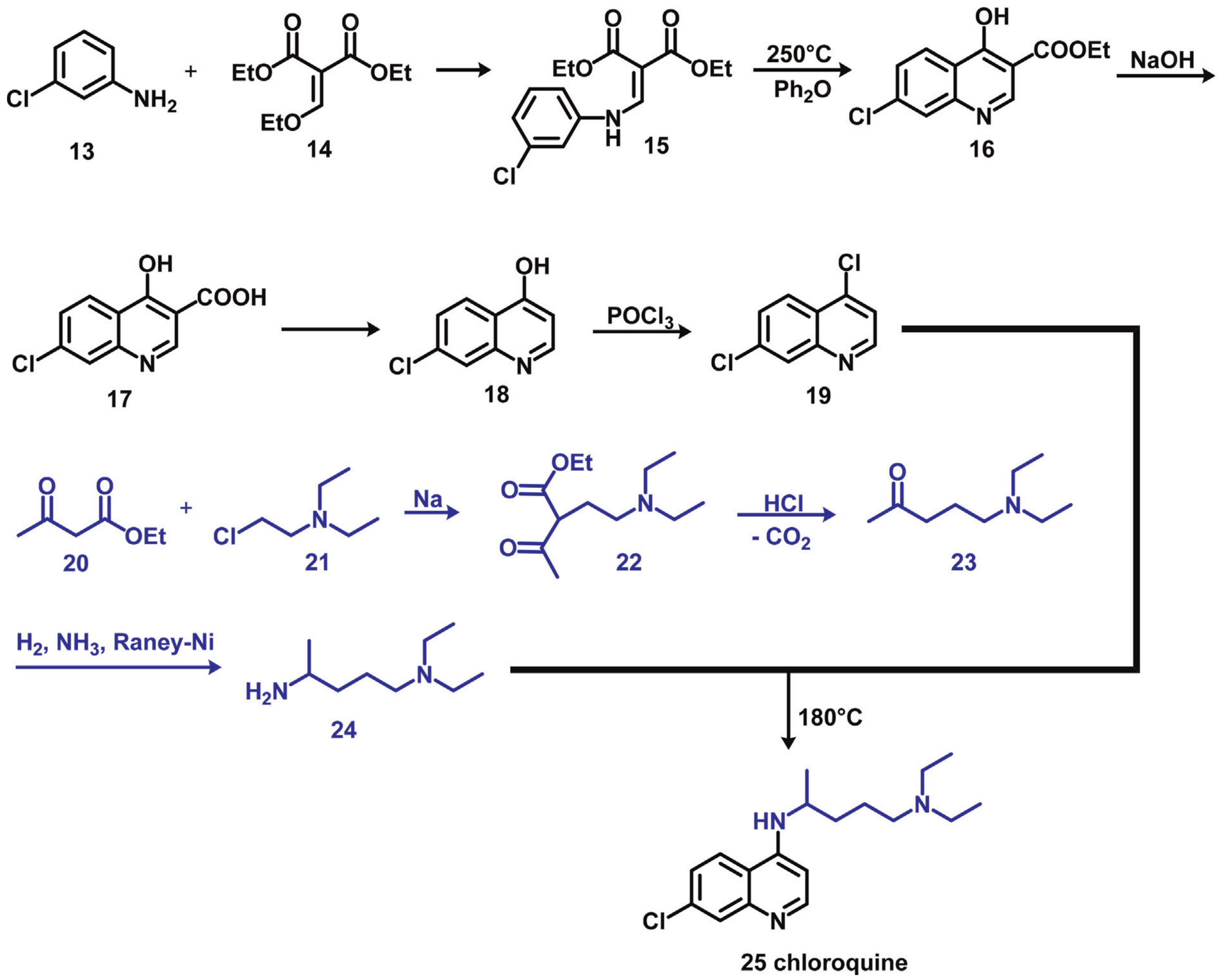


*Fig. 10. Synthesis of chloroquine (CQ).*

compound with hydrogen and ammonia using Raney nickel as a catalyst yielded 24. Nucleophilic aromatic substitution of chlorine at C-4 in 19 with novaldiamine 24 generated the desired molecule chloroquine 25.

Finally, we provide two methods (pathways) for the synthesis of chloroquine (CQ) by Synthia Organic Retrosynthesis Software (Merck, Taiwan) in the Supplementary document.

## 8. Concluding and remarks

This review describes several clinical manifestations of COVID-19, analyses the SARS-CoV-2 genome and outlines the life cycle of SARS-CoV-2. Several methods have been used to examine SARS-CoV-2 infections. The Taiwanese government has established several policies for controlling viral spread. Last but not least, we summarized the therapeutic agents against COVID-19 as mentioned by COVID-19 treatment guidelines.

To avoid direct contact with suspected COVID-19 cases, viral secretions and infected droplets, the following relevant preventive measures should be followed:

(1) Pay attention to and cooperate with the latest epidemic prevention policies issued by the government.
(2) Maintain hand hygiene habits, particularly avoiding touching the eyes, nose and mouth with unclean hands.
(3) Maintain social distancing or wear masks, avoid crowded public places and taking public transportation.
(4) Reduce hospital visits except for urgent medical needs.
(5) Comply with relevant regulations if home quarantined or in isolation.
(6) Stop working or going to school when sick.

(7) Inform your medical providers about your travel history, contact history, occupation and cluster history.

We expect Taiwan to globally interact and cooperate with other countries to develop rapid and accurate screening assays, produce vaccines, design novel agents against SARS-CoV-2 and reduce the side effects. Ultimately, our long-term goal is to be free from COVID-19.

## Authors' contributions

YJC, JHC, FJT and JSY were involved in the conception of this study. CWF, MJH, HAH, SCK, JJL, YNJ, YDC and CCC were involved in the literature search and critical reviewing of the manuscript. SCT, JSL, SCK, FJT and JSY were involved in the preparation of the draft of the manuscript. YJC, JHC, FJT and JSY were involved in the revising and editing of the manuscript. All authors have read and approved the final manuscript.

## Conflict of interest

The authors declare that they have no competing interests.

## Acknowledgements

This work was supported by the project from Dr. Jai-Sing Yang of China Medical University Hospital (DMR-109-147), the Ministry of Science and Technology, Taiwan (MOST 109-2320-B-039-041) and also by the Chinese Medicine Research Center, China Medical University from The Featured Areas Research Center Program within the framework of the Higher Education Sprout Project by the Ministry of Education in Taiwan.

We gratefully acknowledge the support of NVIDIA AI Tech Center for having Clara Discovery and GPU to generate molecular dynamic video on performing the molecular docking to RdRp to our research. We wish to acknowledge the work of Nian-Gu Chen, Pei-Jen Chung for their assistance on this work. We also thank Merck Ltd. Taiwan Company for providing Synthia Organic Retrosynthesis Software (Synthia™).

## Supplementary document. Paths of analysis*

Analysis 2892
Synthia
April 16, 2020

### 1. Analysis parameters

**Analysis type:** Automatic Retrosynthesis
**Rules:** none selected
**Filters:** Cut All Heterocycles, Strategies
**Max. paths returned:** 50
**Max. iterations:** 1000
**Buyable:**

1. Max. molecular weight - 1000 g/mol
2. Max. price - 1000 $/g

**Known:**

1. Max. molecular weight - 1000 g/mol
2. Min. popularity - 25

**Inventory:**

1. Max. molecular weight - 1000 g/mol

**Shorter paths:** no
**Pathway linearity:** COMBO
**Protecting groups:** LESS
**Reaction scoring formula:** 20+1000000*(FILTERS+CONFLICT+NON_SELECTIVITY)+40*PROTECT
**Chemical scoring formula:** SMALLER3, SMALLER1.5
**Min. search width:** 100
**Max. reactions per product:** 20

### 2. Paths

2 paths found. *Paths are sorted by score. Reactions are sorted in appearance order for each path.*

#### *2.1. Path 1*

**Score:** 58.79

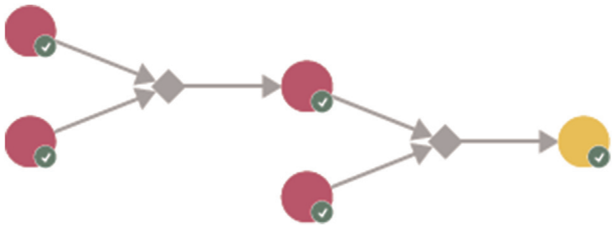

*Figure 1. Outline of path 1.*

* The results stated herein were generated using the proprietary platform owned and maintained by Grzybowski Scientific Inventions, Inc., a subsidiary of Merck KGaA, Darmstadt Germany. The results are provided on an as is basis, and shall be used solely in connection with the rights afforded in the license agreement and for no other purpose.

### 2.1.1. *Synthesis of quinolones*

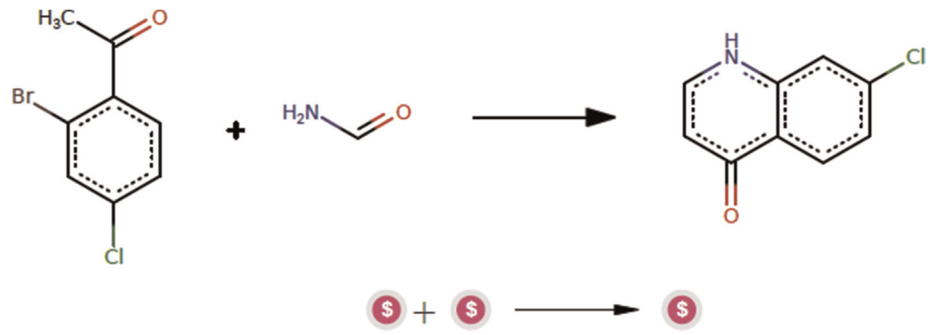

**Substrates:**

1. formamide – *available at Sigma-Aldrich*
2. 2-brom-4-chlor-acetophenon – *available at Sigma-Aldrich*

**Products:**

1. 7-chloro-quinolin-4-ol – *available at Sigma-Aldrich*

**Typical conditions:** [Pd] or [Cu].Cs2CO3.NaOtBu.dioxane
**Protections:** none
**Yield:** good
**Reference:** 10.1055/s-0030-1260198 AND 10.1021/jo701384n AND 10.1021/ol800837z
**Retrosynthesis ID:** 12281

### 2.1.2 *Amination of pyridones*

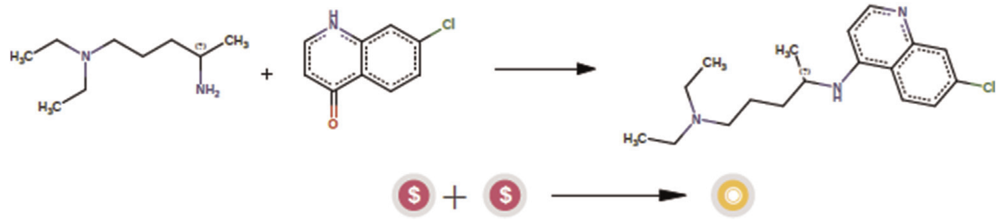

**Substrates:**

1. n4,n4-diaethyl-1-methyl-butandiyldiamin – *available at Sigma-Aldrich*
2. 7-chloro-quinolin-4-ol – *available at Sigma-Aldrich*

**Products:**

1. (-)-chloroquine – *available at Sigma-Aldrich*

**Typical conditions:** 1.PCl5.2.amine
**Protections:** none
**Yield:** good
**Reference:** 10.1021/jm300780p AND 10.3390/molecules170910902 AND 10.1021/jm00392a017
**Retrosynthesis ID:** 14885

### 2.2. *Path 2*

**Score:** 117.57

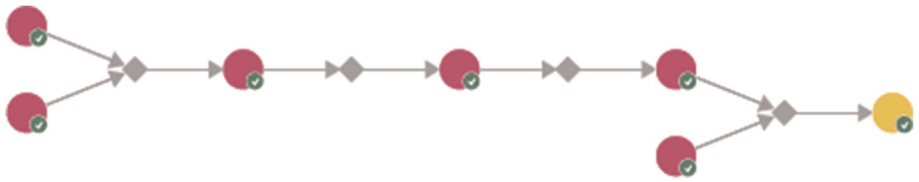

*Figure 2. Outline of path 2.*

### *2.2.1. Synthesis of quinolones*

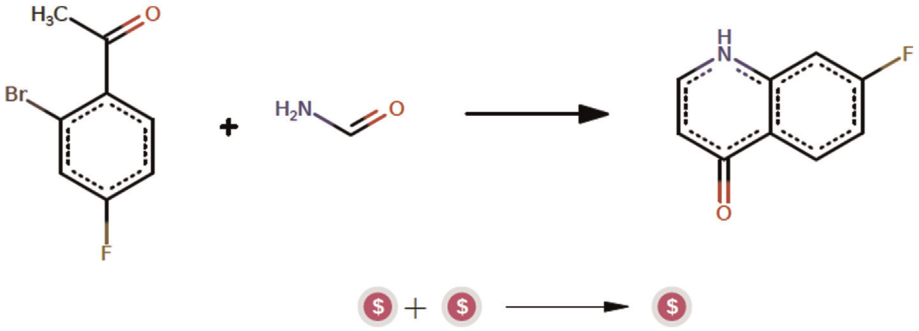


**Substrates:**

1. formamide − *available at Sigma-Aldrich*
2. 2-brom-4-fluor-acetophenon − *available at Sigma-Aldrich*

**Products:**

1. 7-fluoro-quinolin-4-ol − *available at Sigma-Aldrich*

**Typical conditions:** [Pd] or [Cu].Cs2CO3.NaOtBu.dioxane
**Protections:** none
**Yield:** good
**Reference:** 10.1055/s-0030-1260198 AND 10.1021/jo701384n AND 10.1021/ol800837z
**Retrosynthesis ID:** 12281

### *2.2.2. Nucleophilic aromatic substitution*

**Substrates:**

1. 7-fluoro-quinolin-4-ol − *available at Sigma-Aldrich*

**Products:**

1. 7-amino-quinolin-4-ol − *available at Sigma-Aldrich*

**Typical conditions:** Solvent
**Protections:** none
**Yield:** good
**Reference:** 10.1002/9781118093559.ch4
**Retrosynthesis ID:** 49478

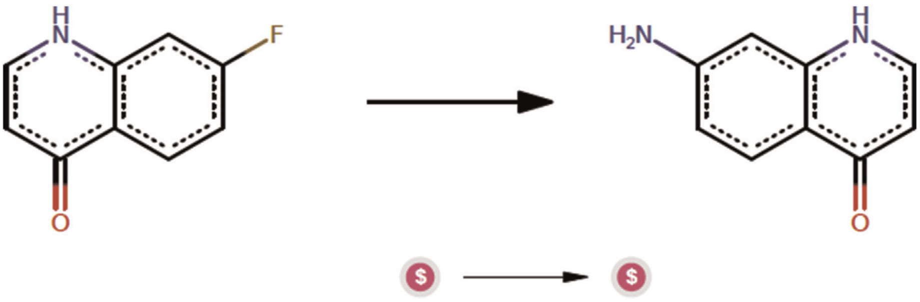

### 2.2.3. *Sandmeyer Reaction*

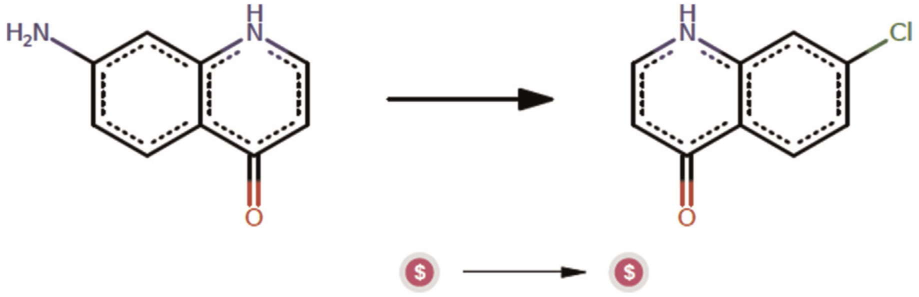


**Substrates:**

1. 7-amino-quinolin-4-ol — *available at Sigma-Aldrich*

**Products:**

1. 7-chloro-quinolin-4-ol — *available at Sigma-Aldrich*

**Typical conditions:** t-BuONO.CuCl2 or TMSCl.MeCN.reflux or NaNO2.Hcl.CuCl
**Protections:** none
**Yield:** moderate
**Reference:** DOI: 10.1016/j.ejmech.2013.01.046 and 10.1021/jm040185a and 10.1021/acs.jmedchem.5b00152 and 10.1021/ja066472g (SI, page S14) and 10.1021/jm3012273 and 10.1002/ejoc.201001395 and 10.1002/ejic.201501400 and 10.1016/j.bmcl.2011.08.073
**Retrosynthesis ID:** 29905

**Substrates:**

1. n4,n4-diaethyl-1-methyl-butandiyldiamin — *available at Sigma-Aldrich*
2. 7-chloro-quinolin-4-ol — *available at Sigma-Aldrich*

**Products:**

1. (-)-chloroquine — *available at Sigma-Aldrich*

**Typical conditions:** 1.PCl5.2.amine
**Protections:** none
**Yield:** good
**Reference:** 10.1021/jm300780p AND 10.3390/molecules170910902 AND 10.1021/jm00392a017
**Retrosynthesis ID:** 14885

### 2.2.4. *Amination of pyridones*

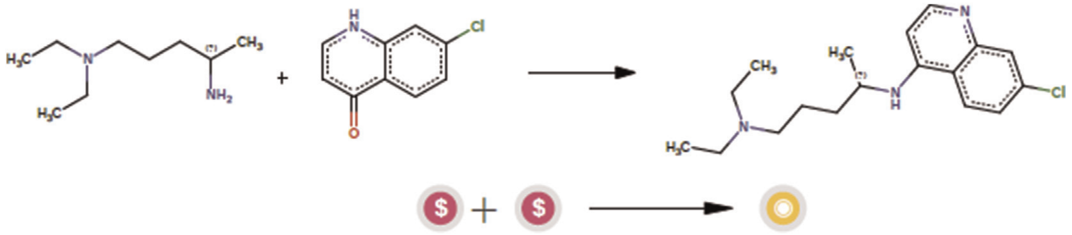

## References


[1] World Health Organization. (WHO) - Clinical management of COVID-19. 2020. Updated Accessed Nov 28th 2020, https://www.who.int/publications/i/item/clinical-management-of-covid-19.

[2] United States National Institutes of Health (NIH) - Coronavirus Disease. 2019 (COVID-19) Treatment Guidelines. 2020. https://www.covid19treatmentguidelines.nih.gov/. [Accessed 18 November 2020].

[3] Haut Conseil de la santé publique - Avis et rapports du HCSP. 2020. https://www.hcsp.fr/explore.cgi/avisrapports. [Accessed 28 November 2020].

[4] China's National Health Commission -. Diagnosis and Treatment Protocol for COVID-19 Treatment. 2020. Updated Sep 01 2020, http://en.nhc.gov.cn/2020-09/01/c_81537.htm. [Accessed 28 November 2020].

[5] Australian National COVID-19 Clinical Evidence Taskforce - Australian guidelines for the clinical care of people with COVID-19. 2020. Updated Nov 26 2020, https://app.magicapp.org/#/guideline/4632. [Accessed 28 November 2020].

[6] Yang J-S, Chiang J-H, Tsai SC, Hsu Y-M, Bau D-T, Lee K-H, et al. In Silico De Novo Curcuminoid Derivatives From the Compound Library of Natural Products Research Laboratories Inhibit COVID-19 3CLpro Activity. Natural Product Communications 2020;15. 1934578X20953262.

[7] Chang YT, Lin CY, Tsai MJ, Hung CT, Hsu CW, Lu PL, et al. Infection control measures of a Taiwanese hospital to confront the COVID-19 pandemic. Kaohsiung J Med Sci 2020;36:296–304.

[8] Yip PS, Hsieh YH, Xu Y, Lam KF, King CC, Chang HL. Assessment of intervention measures for the 2003 SARS epidemic in Taiwan by use of a back-projection method. Infect Control Hosp Epidemiol 2007;28:525–30.

[9] Chen CC, Tseng CY, Choi WM, Lee YC, Su TH, Hsieh CY, et al. Taiwan Government-Guided Strategies Contributed to Combating and Controlling COVID-19 Pandemic. Front Public Health 2020;8:547423.

[10] Chen SF, Huang LH, Chen CM, Chuang TH, Peng MT, Wang HH. [The Key Role of Taiwanese Nurses in Combating COVID-19 Pandemic]. Hu Li Za Zhi 2020;67:84–9.

[11] Tsai SC, Lu CC, Bau DT, Chiu YJ, Yen YT, Hsu YM, et al. Approaches towards fighting the COVID-19 pandemic (Review). Int J Mol Med 2021;47:3–22.

[12] Biko DM, Ramirez-Suarez KI, Barrera CA, Banerjee A, Matsubara D, Kaplan SL, et al. Imaging of children with COVID-19: experience from a tertiary children's hospital in the United States. Pediatr Radiol 2020;18:1–9.

[13] Lu J, Zhang Y, Cheng G, He J, Wu F, Hu H, et al. Clinical characteristics and outcomes of adult critically ill patients with COVID-19 in Honghu, Hubei Province. Nan Fang Yi Ke Da Xue Xue Bao 2020;40:778–85.

[14] Wang A, Gao G, Wang S, Chen M, Qian F, Tang W, et al. Clinical Characteristics and Risk Factors of Acute Respiratory Distress Syndrome (ARDS) in COVID-19 Patients in Beijing, China: A Retrospective Study. Med Sci Monit 2020;26:e925974.

[15] Bonetti G, Manelli F, Patroni A, Bettinardi A, Borrelli G, Fiordalisi G, et al. Laboratory predictors of death from coronavirus disease 2019 (COVID-19) in the area of Valcamonica, Italy. Clin Chem Lab Med 2020;58:1100–5.

[16] Bolondi G, Russo E, Gamberini E, Circelli A, Meca MCC, Brogi E, et al. Iron metabolism and lymphocyte characterisation during Covid-19 infection in ICU patients: an observational cohort study. World J Emerg Surg 2020;15:41.

[17] Elamari S, Motaib I, Zbiri S, Elaidaoui K, Chadli A, Elkettani C. Characteristics and outcomes of diabetic patients infected by the SARS-CoV-2. Pan Afr Med J 2020;37:32.

[18] Li J, Yan R, Zhai Y, Qi X, Lei J. Chest CT findings in patients with coronavirus disease 2019 (COVID-19): a comprehensive review. Diagn Interv Radiol 2020;1:1–12.

[19] Chen X, Zhang G, Hao SY, Bai L, Lu JJ. Similarities and Differences of Early Pulmonary CT Features of Pneumonia Caused by SARS-CoV-2, SARS-CoV and MERS-CoV: Comparison Based on a Systemic Review. Chin Med Sci J 2020;35:254–61.

[20] Zhao Y, Liu X, Duan W, Liu Y, Du W, Du Y, et al. A typical computed tomography presentations of coronavirus disease 2019. Radiol Infect Dis 2020;7:130–4.

[21] Wang YC, Lee YT, Yang T, Sun JR, Shen CF, Cheng CM. Current diagnostic tools for coronaviruses-From laboratory diagnosis to POC diagnosis for COVID-19. Bioeng Transl Med 2020;13:5(3):e10177:1–10.

[22] Shih HI, Wu CJ, Tu YF, Chi CY. Fighting COVID-19: A quick review of diagnoses, therapies, and vaccines. Biomed J 2020;43:341–54.

[23] Jing R, Kudinha T, Zhou ML, Xiao M, Wang H, Yang WH, et al. Laboratory diagnosis of COVID-19 in China: A review of challenging cases and analysis. J Microbiol Immunol Infect 2020;17(20). 30249-8.

[24] Luo YH, Chiu HY, Weng CS, Chen YM. Overview of coronavirus disease 2019: Treatment updates and advances. J Chin Med Assoc 2020;83:805–8.

[25] Rawat K, Kumari P, Saha L. COVID-19 vaccine: A recent update in pipeline vaccines, their design and development strategies. Eur J Pharmacol 2020;25(892):173751–604.

[26] Li Q, Guan X, Wu P, Wang X, Zhou L, Tong Y, et al. Early Transmission Dynamics in Wuhan, China, of Novel Coronavirus-Infected Pneumonia. N Engl J Med 2020;382:1199–207.

[27] Kupferschmidt K, Cohen J. Race to find COVID-19 treatments accelerates. Science 2020;367:1412–3.

[28] Chu WM, Shieh GJ, Wu SL, Sheu WH. Use of Facebook by Academic Medical Centers in Taiwan During the COVID-19 Pandemic: Observational Study. J Med Internet Res 2020;22:e21501.

[29] Lin CH, Lin YW, Wang JY, Lin MH. The pharmaceutical practice of mask distribution by pharmacists in Taiwan's community pharmacies under the Mask Real-Name System, in response to the COVID-19 outbreak. Cost Eff Resour Alloc 2020;18:45.

[30] Hirschmann MT, Hart A, Henckel J, Sadoghi P, Seil R, Mouton C. COVID-19 coronavirus: recommended personal protective equipment for the orthopaedic and trauma surgeon. Knee Surg Sports Traumatol Arthrosc 2020;28(6):1690–8.

[31] Saadat S, Rawtani D, Hussain CM. Environmental perspective of COVID-19. Sci Total Environ 2020;728:138870.

[32] Bartoszko JJ, Farooqi MAM, Alhazzani W, Loeb M. Medical masks vs N95 respirators for preventing COVID-19 in healthcare workers: A systematic review and meta-analysis of randomized trials. Influenza Other Respir Viruses; 2020.

[33] Ma QX, Shan H, Zhang HL, Li GM, Yang RM, Chen JM. Potential utilities of mask-wearing and instant hand hygiene for fighting SARS-CoV-2. J Med Virol 2020;92(9):1567–71.

[34] Long Y, Hu T, Liu L, Chen R, Guo Q, Yang L, et al. Effectiveness of N95 respirators versus surgical masks against influenza: A systematic review and meta-analysis. J Evid Based Med 2020;13(2):93–101.

[35] Ziegler CGK, Allon SJ, Nyquist SK, Mbano IM, Miao VN, Tzouanas CN, et al. SARS-CoV-2 Receptor ACE2 Is an Interferon-Stimulated Gene in Human Airway Epithelial Cells and Is Detected in Specific Cell Subsets across Tissues. Cell. 2020.

[36] Zhao B, Liu S, Liu Y, Li G, Zhang Q. [Liquid chromatography tandem mass spectrometry for therapeutic drug monitoring of voriconazole in heat-inactivated blood samples: its application during COVID-19 pandemic]. Nan Fang Yi Ke Da Xue Xue Bao 2020;40:342–5.

[37] Brewer C, Streel E. Is Alcohol in Hand Sanitizers Absorbed Through the Skin or Lungs? Implications for Disulfiram Treatment. Alcohol Alcohol 2020;55(4):354–6.

[38] Henwood AF. Coronavirus disinfection in histopathology. J Histotechnol 2020:1–3.
[39] Kampf G, Todt D, Pfaender S, Steinmann E. Persistence of coronaviruses on inanimate surfaces and their inactivation with biocidal agents. J Hosp Infect 2020;104:246–51.
[40] Chen C, Zhang XJ, Wang Y, Zhu LX, Liu J. Waste water disinfection during SARS epidemic for microbiological and toxicological control. Biomed Environ Sci 2006;19:173–8.
[41] Jones L, Walsh K, Willcox M, Morgan P, Nichols J. The COVID-19 pandemic: Important considerations for contact lens practitioners. Cont Lens Anterior Eye 2020;43:196–203.
[42] Torres AE, Lyons AB, Narla S, Kohli I, Parks-Miller A, Ozog D, et al. Ultraviolet-C and other methods of decontamination of filtering facepiece N-95 respirators during the COVID-19 pandemic. Photochem Photobiol Sci 2020;19:746–51.
[43] Caruso AA, Del Prete A, Lazzarino AI, Capaldi R, Grumetto L. May hydrogen peroxide reduce the hospitalization rate and complications of SARS-CoV-2 infection? Infect Control Hosp Epidemiol 2020:1–5.
[44] Cheng VCC, Wong SC, Kwan GSW, Hui WT, Yuen KY. Disinfection of N95 respirators by ionized hydrogen peroxide during pandemic coronavirus disease 2019 (COVID-19) due to SARS-CoV-2. J Hosp Infect 2020;105(2):358–9.
[45] Schrank CL, Minbiole KPC, Wuest WM. Are Quaternary Ammonium Compounds, the Workhorse Disinfectants, Effective against Severe Acute Respiratory Syndrome-Coronavirus-2? ACS Infect Dis 2020;6(7):1553–7.
[46] Verbeek JH, Rajamaki B, Ijaz S, Sauni R, Toomey E, Blackwood B, et al. Personal protective equipment for preventing highly infectious diseases due to exposure to contaminated body fluids in healthcare staff. Cochrane Database Syst Rev 2020;4:CD011621.
[47] Caruso AA, Del Prete A, Lazzarino AI, Capaldi R, Grumetto L. Might hydrogen peroxide reduce the hospitalization rate and complications of SARS-CoV-2 infection? Infect Control Hosp Epidemiol 2020;41:1360–1.
[48] Wang CC, Chao JK, Wang ML, Yang YP, Chien CS, Lai WY, et al. Care for Patients with Stroke During the COVID-19 Pandemic: Physical Therapy and Rehabilitation Suggestions for Preventing Secondary Stroke. J Stroke Cerebrovasc Dis 2020;29:105182.
[49] Wang CC, Chao JK, Chang YH, Chou CL, Kao CL. Care for patients with musculoskeletal pain during the COVID-19 pandemic: Physical therapy and rehabilitation suggestions for pain management. J Chin Med Assoc 2020;83:822–4.
[50] Sankar PR, Viswanath A, Babu PS, Supriya K. Potential anti-COVID-19 drug options. Int J Pharm Sci Rev Res 2020;62:199–204.
[51] Fan L, Jiang S, Yang X, Wang Z, Yang C. COVID-19 Drug Treatment in China. Curr Pharmacol Rep 2020;6:146–54.
[52] Kumar P, Sah AK, Tripathi G, Kashyap A, Tripathi A, Rao R, et al. Role of ACE2 receptor and the landscape of treatment options from convalescent plasma therapy to the drug repurposing in COVID-19. Mol Cell Biochem 2020. Ahead of Print.
[53] Chibber P, Haq SA, Ahmed I, Andrabi NI, Singh G. Advances in the possible treatment of COVID-19: A review. Eur J Pharmacol 2020;15(883):173372–87.
[54] Siegel D, Hui HC, Doerffler E, Clarke MO, Chun K, Zhang L, et al. Discovery and Synthesis of a Phosphoramidate Prodrug of a Pyrrolo[2,1-f][triazin-4-amino] Adenine C-Nucleoside (GS-5734) for the Treatment of Ebola and Emerging Viruses. J Med Chem 2017;60:1648–61.
[55] ~~Drake NL, Creech HJ, et al.~~ Synthetic antimalarials; the preparation and properties of 7-chloro-4-(4-diethylamino-l-methylbutylamino)-quinoline (SN-7618). J Am Chem Soc 1946;68:1214–6.
[56] Price CC, Roberts RM. The synthesis of 4-hydroxyquinolines; through ethoxymethylene malonic ester. J Am Chem Soc 1946;68:1204–8.

Drake NL, Creech HJ, Draper D, Garman JA, Haywood S, Peck RM, et al.
