# Supplementary material for: Analysis of COVID-19 prevention and treatment in Taiwan
Source: Biomedicine (Taipei). 2021 Mar 1;11(1):1–18. doi: 10.37796/2211-8039.1185 (PMC8823471; doi:10.37796/2211-8039.1185)
Supplement: Supplementary file 2 [file bmed-11-01-001-s002.docx]

**Dear editors of BioMedicine:**

We have made corrections and attached please find the revised the proofs of our article (**Manuscript No.: MS #1185**). If you need any further information regarding our manuscript, please let me know.

Sincerely yours,

Research Fellow

Jai-Sing Yang Ph.D

Department of Medical Research, China Medical University Hospital, China Medical University, 2, Yuh-Der Rd., Taichung 40447,Taiwan.Telephone: +886-4-22052121-4125

2021/01/11
